# Healthcare Practitioners' Quality of Life in Rural and Urban Areas of Saudi Arabia

**DOI:** 10.7759/cureus.46712

**Published:** 2023-10-09

**Authors:** Nada M Allhaiby, Asia M Kalantan, Mariam M Tunkar, Maha A Abuzenada, Basim S Alsaywid

**Affiliations:** 1 Medicine and Surgery, Umm Al-Qura University, Makkah, SAU; 2 Medicine and Surgery, King Saud bin Abdulaziz University for Health Sciences (KSAU-HS), Jeddah, SAU; 3 Research and Development, Saudi Commission for Health Specialties, Jeddah, SAU; 4 Education and Research Skills, Saudi National Institute of Health, Riyadh, SAU

**Keywords:** saudi arabia, urban, rural, quality of life, healthcare practitioners

## Abstract

Introduction: The quality of care delivered by healthcare practitioners (HCPs) is crucial in promoting optimal health and quality of life (QOL) for a population. To achieve this, understanding the factors that affect the quality of life of healthcare practitioners is essential for governments to develop sustainable healthcare systems. Developed countries have a major role to play in this aspect, as the misallocation of healthcare providers to the wrong geographic regions can significantly impact their performance.

Aim: This study aims to evaluate the factors associated with healthcare practitioners’ (HCP) quality of life (QOL) and provide workforce planning with knowledge of the level of QOL among HCPs and its factors in Saudi Arabia in 2021.

Methods: This is an observational, descriptive, cross-sectional study conducted in both rural and urban areas of Saudi Arabia. The study population includes all healthcare practitioners practicing in Saudi Arabia. A probability-stratified random sampling technique was used to recruit healthcare practitioners into the study, with a requirement of at least 380 practitioners to achieve 95% confidence and a 5% margin of error. To assess the quality of life of healthcare practitioners in Saudi Arabia, the study used a national online self-administered questionnaire that was designed by the research team. The data collection process took place from June 2021 to October 2021, and responses were obtained randomly. For analysis, the study used descriptive statistics such as frequency, percentages, mean or median, and standard deviation or interquartile range. The statistical significance was set at p<0.05, and independent sample T-tests and Chi-square tests were calculated to determine any significant differences between groups.

Results: A total of 439 participants completed the questionnaire and were included in the final analysis. The participants had a mean age of 38.8 years (SD = 10.173), with 232 (52.8%) male and 207 (47.2%) female. Regarding marital status, 28% were single, 68.6% were married, and 3.4% were divorced or widowed. The prevalence of chronic diseases in the cohort was 9.1%, with hypertension being the most commonly reported. Of the participants, 362 (82.5%) were living in a society considered urban, while 77 (17.5%) were living in rural areas. Urban healthcare practitioners expressed higher levels of satisfaction with safety and security, internet availability and speed, and city infrastructure compared to their rural counterparts. However, rural practitioners reported greater satisfaction with the cost of living, and transportation quality was a point of concern for both groups.

Conclusion: The study shows that people living in urban and rural areas are all satisfied with their living conditions based on many factors mentioned in the results section. This indicates that there is no significant difference. The most important factor that affects satisfaction with living is health status. The rate of satisfaction is very high for all factors, including security and safety, environmental health, city infrastructure, cost of living, internet availability, and sports activity-all of which are related to the city itself. For factors related to the individual, such as emotional support from family and friends, personal relationships, overall health, and body appearance, the rate of satisfaction is also high.

## Introduction

The standard of care provided by healthcare practitioners (HCPs) is essential in promoting optimal health and quality of life for a population. Therefore, it is vital that HCPs lead healthy and high-quality lifestyles themselves. In addition, HCPs play an integral role in both pharmacological and non-pharmacological approaches to treating a patient's condition or disease, from developing an effective and safe treatment plan to delivering psychological support to their patients [1].

The World Health Organization (WHO) defines quality of life (QOL) as a complex, multidimensional concept that encompasses individual and environmental factors, culture and values, one's position within it, as well as goals, expectations, values, and worries [2]. Rural areas are characterized by spacious land with fewer people and homes, in contrast to urban areas with high-density housing and infrastructure [3,4]. Despite this, significant differences exist concerning the quality of life of healthcare practitioners in rural versus urban areas.

Having an accurate understanding of the quality of life of healthcare practitioners is essential for governments to create pathways toward community development. Developed countries play a major role in this area, and there are many factors that can influence their quality of life. For example, the misallocation of healthcare providers to inappropriate geographic regions can have dramatic impacts on performance, such as in Canada, where an imbalance in the distribution of healthcare services has resulted in an increased workload for medical teams, leading to added stress, strain, and overworking [5]. In 2016, there were 84,063 physicians and 396,249 nurses in Canada; therefore, effective reallocation of these two groups should have positive impacts on any policy interventions. Unfortunately, practitioners choose to work and live in urban areas rather than rural ones, creating the challenge of ensuring that an adequate number of each profession is present in different communities and avoiding underemployed or underserved areas [5].

Many experienced healthcare practitioners, such as nurses, have been migrating to developed countries in increasing numbers due to a desire for greater personal and professional development, improved quality of life, and increased safety. Additionally, they seek better salaries, training opportunities, and the prospect of their skills being highly valued and better utilized [6,7]. However, this migration has resulted in a shortage of healthcare staff in their countries of origin. As a result, the remaining nurses and healthcare practitioners have to handle an increased workload, leading to higher stress levels [8].

The significance of our study is to provide a comprehensive understanding of healthcare practitioners from both rural and urban areas in the Kingdom of Saudi Arabia (KSA). This study aims to collect the necessary statistical data to aid workforce planning, improve their quality of life, and identify various factors and aspects affecting it.

## Materials and methods

Study design and setting

This was an observational, descriptive, cross-sectional study conducted in many rural and urban areas in Saudi Arabia.

Study population, sample size calculation, and sampling technique

The inclusion criteria were that all healthcare practitioners live in Saudi Arabia. A probability-stratified random sampling technique was used to recruit healthcare practitioners into the study. The calculated sample size was 380 participants to be 95% confident with five margins of error in a population size of 690,000 registered practitioners.

Research instrument

We employed a national online self-administered questionnaire to randomly obtain data from all healthcare practitioners in Saudi Arabia in order to assess their quality of life. The questionnaire was designed by the research team with an academic lecturer, and corrections were made based on the comments received. The data collection process took place from June 2021 to October 2021.

Our self-report instrument consisted of a consent form and sociodemographic profile that included age, gender, job title, marital status, health status, address, and whether the area was considered a rural or urban area. It also included 21 short-form multiple-choice questions on quality of life as well as two open-ended questions to identify the factors that affect the quality of life of healthcare practitioners in different rural and urban areas in Saudi Arabia.

Data analysis

Simple descriptive statistics were reported as frequency and percentage for qualitative data, mean with standard deviation for quantitative normally distributed data or median and interquartile range for quantitative skewed data. For bivariate analysis, with a two-tailed statistical significance set at p = 0.05, an independent sample, student t-test, and chi-square test were calculated to detect the significance between study factors and outcome factors.

Selection and information bias have been minimized as much as possible. Data entry was done in Microsoft Excel (Microsoft Corporation, New York, USA), and outcomes were recorded and analyzed.

Ethical consideration

To ensure the ethical validity of the study, we obtained approval from the Ethical Committee of the Research and Development Department at the Saudi Commission for Health Specialties. All participating healthcare practitioners were required to provide written informed consent prior to filling out the survey. No personal information was collected, and all collected data were treated with strict confidentiality measures in place to protect participants’ privacy. Additionally, participants were informed of their right to withdraw from the study at any time without penalty, and any questions or concerns regarding the study were addressed by the research team promptly and professionally.

## Results

A total of 439 participants completed the questionnaire and were included in the final analysis. The mean age was 38.8 years (SD = 10.173), and 232 (52.8%) were male, while 207 (47.2%) were female. In terms of marital status, 28% were single, 68.6% were married, and 3.4% were divorced or widowed. The prevalence of chronic diseases among the participants was 9.1%, with hypertension being the most common chronic illness reported. Of these participants, 362 (82.5%) lived in urban areas, while 77 (17.5%) lived in rural areas (Table 1).

**Table 1:** Shows the basic demographic differences between those living in urban and rural communities.

Factor	Urban group (n = 362)	Rural group (n = 77)	Statistics
Gender	Male = 178 (49.2%), Female = 184 (50.8%)	Male = 54 (70.1%), Female = 23 (29.9%)	Chi-square = 11.193, df = 1, p = 0.001
Age in years	Mean = 39.1 years (SD = 10.36)	Mean = 37.7 years (SD = 9.202)	t = –1.087, p = 0.277
Marital status	Single = 30%, Married = 66%, Divorced or widower = 4%	Single = 18%, Married = 82%, Divorced or Widower = 0%	Chi-square = 8.807, df = 2, p = 0.012
Prevalence of chronic illness	10.2%	3.9%	Chi-square = 3.067, df = 1, p = 0.05

Quality of life variations in healthcare practitioners

According to the survey results on safety and security among healthcare practitioners, urban practitioners (77%) reported higher levels of satisfaction compared to rural practitioners (57%). This difference was statistically significant with a P-value of 0.001. In terms of cost of living, 39% of urban practitioners were satisfied, whereas 72% of rural practitioners reported satisfaction (P = 0.009). For internet availability and speed, 10.3% of practitioners reported poor levels of satisfaction, while 31.9% reported moderate levels and 57.8% reported good to excellent levels (P = 0.004). City crowdedness was rated as not good by 24.4%, not bad by 43.3%, and good or excellent by 32.3% of respondents, with a statistically significant P-value of 0.001. The majority of participants (56.3%) rated city infrastructure as good or excellent. Finally, 60% of urban practitioners reported satisfaction with the quality of transportation, compared to only 53% of rural practitioners who reported dissatisfaction. For more detailed information, see Table 2.

**Table 2:** Quality of life questionnaire among healthcare practitioners in rural and urban societies in Saudi Arabia.

Question	Overall	Urban (n = 362)	Rural (n = 77)	Statistics
Safety and security	Satisfied = 73.1%, Not satisfied = 26.9%	Satisfied = 77%, Not satisfied = 23%	Satisfied = 57%, Not satisfied = 43%	Chi-square = 12.129, df = 1, p = 0.001
City environmental health	Not good = 8.4%, Not bad = 31.2%, Good or excellent = 60.4%	Not good = 9%, Not bad = 31%, Good or excellent = 60%	Not good = 5%, Not bad = 33%, Good or excellent = 62%	Chi-square = 1.26, df = 2, p = 0.53
City infrastructure (streets and buildings)	Not good = 10.7%, Not bad = 33%, Good or excellent = 56.3%	Not good = 9%, Not bad = 32%, Good or excellent = 59%	Not good = 17%, Not bad = 40%, Good or excellent = 43%	Chi-square = 7.789, df = 2, p = 0.02
Cost of living	Satisfied = 41.7%, Not satisfied = 58.3%	Satisfied = 39%, Not satisfied = 61%	Satisfied = 55%, Not satisfied = 45%	Chi-square = 6.353, df = 1, p = 0.009
Internet availability and speed	Not good = 10.3%, Not bad = 31.9%, Good or excellent = 57.8%	Not good = 9%, Not bad = 30%, Good or excellent = 61%	Not good = 18%, Not bad = 39%, Good or excellent = 43%	Chi-square = 10.826, df = 2, p = 0.004
Quality of transportation	Satisfied = 58.1%, Not satisfied = 41.9%	Satisfied = 60%, Not satisfied = 40%	Satisfied = 47%, Not satisfied = 53%	Chi-square = 4.926, df = 1, p = 0.019
City crowdedness	Not good = 24.4%, Not bad = 43.3%, Good or excellent = 32.3%	Not good = 27%, Not bad = 44%, Good or excellent = 29%	Not good = 13%, Not bad = 38%, Good or excellent = 49%	Chi-square = 13.995, df = 2, p = 0.001
Quality of sport activity	Not good = 26.4%, Not bad = 41.7%, Good or excellent = 31.9%	Not good = 26%, Not bad = 42%, Good or excellent = 32%	Not good = 30%, Not bad = 38%, Good or excellent = 32%	Chi-square = 0.792, df = 2, p = 0.67
Support from others	Not much = 34.2%, Sometimes = 35.8%, Most of times = 30%	Not much = 32%, Sometimes = 38%, Most of times = 30%	Not much = 43%, Sometimes = 25%, Most of times = 32%	Chi-square = 5.452, df = 2, p = 0.065
Support from friends	Satisfied = 62%, Not satisfied = 38%	Satisfied = 64%, Not satisfied = 36%	Satisfied = 53%, Not satisfied = 47%	Chi-square = 3.007, df = 1, p = 0.055
Personal relationships	Satisfied = 70.4%, Not satisfied = 29.6%	Satisfied = 71%, Not satisfied = 29%	Satisfied = 68%, Not satisfied = 32%	Chi-square = 0.365, df = 1, p = 0.317
Self-satisfaction	Satisfied = 76.1%, Not satisfied = 23.9%	Satisfied = 77%, Not satisfied = 23%	Satisfied = 74%, Not satisfied = 26%	Chi-square = 0.217, df = 1, p = 0.369
Own health	Satisfied = 67.7%, Not satisfied = 32.3%	Satisfied = 69%, Not satisfied = 31%	Satisfied = 62%, Not satisfied = 38%	Chi-square = 1.206, df = 1, p = 0.167
Own body appearance	Satisfied = 57.6%, Not satisfied = 42.4%	Satisfied = 57%, Not satisfied = 43%	Satisfied = 62%, Not satisfied = 38%	Chi-square = 0.847, df = 1, p = 0.214
Ability to perform daily living activities	Satisfied = 68.6%, Not satisfied = 31.4%	Satisfied = 68%, Not satisfied = 32%	Satisfied = 70%, Not satisfied = 30%	Chi-square = 0.106, df = 1, p = 0.429
Quality of sex life	Satisfied = 57.2%, Not satisfied = 42.8%	Satisfied = 56%, Not satisfied = 44%	Satisfied = 64%, Not satisfied = 36%	Chi-square = 1.592, df = 1, p = 0.128
Quality of sleep	Satisfied = 55.4%, Not satisfied = 42.8%	Satisfied = 55%, Not satisfied = 45%	Satisfied = 56%, Not satisfied = 44%	Chi-square = 0.009, df = 1, p = 0.513
Negative feeling	Never = 18.5%, Sometimes = 61%, Usually = 12.8%, Always = 7.7%	Never = 17%, Sometimes = 63%, Usually = 13%, Always = 7%	Never = 25%, Sometimes = 52%, Usually = 12%, Always = 11%	Chi-square = 5.186, df = 3, p = 0.159
Access to health services	Satisfied = 59.2%, Not satisfied = 40.8%	Satisfied = 60%, Not satisfied = 40%	Satisfied = 57%, Not satisfied = 43%	Chi-square = 0.168, df = 1, p = 0.387
Local environment	Satisfied = 56.3%, Not satisfied = 43.7%	Satisfied = 57%, Not satisfied = 43%	Satisfied = 55%, Not satisfied = 45%	Chi-square = 0.112, df = 1, p = 0.416
Workplace	Satisfied = 55.1%, Not satisfied = 44.9%	Satisfied = 54%, Not satisfied = 46%	Satisfied = 60%, Not satisfied = 40%	Chi-square = 0.804, df = 1, p = 0.221

## Discussion

The quality of life for healthcare practitioners is a complex, multidimensional concept that depends on several factors. These factors vary according to an individual's culture, environment, and personality. Consequently, having an accurate understanding of their quality of life is essential for governments to open up pathways to community development. According to our study results, a total of 439 healthcare practitioners from rural and urban areas of KSA responded to the survey, and the results revealed that the average quality of life of healthcare practitioners was similar in both urban and rural areas.

The study findings demonstrated that people living in rural or urban areas were satisfied with their living conditions based on various factors mentioned in the results. The most critical factor influencing the satisfaction of life was found to be one's health status, while gender and marital status were deemed less important. Healthcare practitioners expressed high levels of satisfaction with various factors related to the city itself, such as security and safety, environmental health, infrastructure, cost of living, internet availability, and sports activity. Similarly, factors related to the person, such as emotional support from friends and family, personal relationships, overall health, and body appearance, also scored high levels of satisfaction.

A similar study conducted to assess the quality of life of nurses employed at King Abdulaziz University Hospital, Jeddah, revealed that the majority of nurses described their quality of life as either very good or good. However, the study also suggested that certain modifications, such as increasing salaries and reducing the length of shifts, could improve the quality of life of nurses [9]. Another cross-sectional study conducted among hospital personnel in Al-Kharj found that the majority of healthcare practitioners described their quality of life as generally good. The highest scores were associated with those healthcare practitioners working shorter hours, with a less taxing workload, and better job satisfaction [10].

Another study done on nurses to assess the quality of work life conducted across 143 primary healthcare centers in Jazan revealed that nurses who scored below the average of Brook's survey were mostly unsatisfied due to various factors. These included inconvenient and long working hours, lack of necessary facilities, insufficient payment, negative societal portrayals of the nursing profession, and unsuitable working environments. Despite expressing satisfaction with co-workers, many of these nurses still intend to leave their jobs in pursuit of a better quality of life, also showed that factors affecting nurses' quality of life include long working hours, deficient facilities, work-family imbalance, insufficient vacations, inadequate staffing, management and supervisory skills, lack of professional development opportunities, and an unhealthy working environment [11].

Further research has indicated that payment, job safety, career improvement, and the work environment have a direct and strong correlation with the different aspects of the quality of working life in a Dawadmi public hospital. However, healthcare practitioners' performance in their jobs has been found to be significantly impacted on a spiritual level due to these quality-of-working-life factors [12].

In Madinah, a study conducted on nurses' quality of working life suggested that they generally experience a moderate level of quality of life while working. Those who scored higher on the quality of working life scale tended to be non-Saudis, married individuals, those with more work experience, and those working full-time [13]. For the Eastern Province in Saudi Arabia, a study that explored the quality of nurses’ work life demonstrated a significant correlation between its three core elements: nurses’ demographic factors, quality of nurses’ work life, and turnover intention. It was found that the higher the quality of life, the more the decreased likelihood of an intention to leave the job was noted [14].

Furthermore, Korea has been struggling to ensure its rural residents have access to adequate medical care. In order to improve the medical services and environment for healthcare practitioners, the government has been attempting to build stronger relationships between healthcare practitioners and the local communities in order to enhance communication skills. Additionally, there is a clear link between job satisfaction and quality of life for healthcare practitioners; if they feel motivated and engaged, it will have a positive effect on their lives. Quality of life also relies heavily on the sense of belonging one feels in the workplace, as well as cultural differences that may be present in the working environment [15]. There are many factors that can influence the quality of life in developing countries, such as financial incentives and career development. However, for healthcare practitioners, showing support and recognition can be the most effective way to ensure motivation and job satisfaction [16].

Developing countries often suffer from a lack of healthcare practitioners, which leads to understaffed and fragile health systems. For example, it has been estimated that an additional million healthcare workers are needed in Africa to provide sufficient basic services [17]. Furthermore, with the increase in international migration, more healthcare workers are leaving for developed countries [18]. In Brazil, healthcare professionals frequently experience high levels of strain or overwork, which can lead to muscular pain caused by the overuse of muscles or exposure to sunlight [19]. Uganda, another developing African country, has a critical shortage of healthcare practitioners. According to the Uganda Bureau of Statistics (UBOS 2008), 85% of the population resides in rural areas, with low-quality work-related life for healthcare practitioners [20]. In Pakistan, however, a study found that healthcare practitioners were highly satisfied with their health-related quality of life (HR-QOL) in the social domain, though not as much in the physical and environmental domains [21].

Quality of life studies have focused on several factors relating to healthcare practitioners' work, such as job satisfaction, work environment, compensation, social relationships, and managing relationships. Researchers have found that participants usually cited family as the main reason they continue to reside in a specific area. This was followed by work and salary, services, safety and city environment, social restrictions, social life, traffic, crowdedness, cost of living, job opportunities, self-development, entertainment, education, and transportation [22].

Limitations

This study has some notable limitations that should be acknowledged. Firstly, its focus on Saudi Arabia may not account for factors and variations in other cultural contexts or populations. Additionally, the inclusion of solely qualitative studies might be insufficient to conclude a quantitative synthesis. Secondly, the self-administered online questionnaire approach could increase the probability of recall bias, which might compromise the validity of the results. Therefore, it's essential to consider these limitations when interpreting the findings.

## Conclusions

The study shows that people living in urban and rural areas are all satisfied with their living conditions based on many factors mentioned in the results section. This indicates that there is no significant difference. The most important factor that affects satisfaction with living is health status. The rate of satisfaction is very high for all factors, including security and safety, environmental health, city infrastructure, cost of living, internet availability, and sports activity-all of which are related to the city itself. For factors related to the individual, such as emotional support from family and friends, personal relationships, overall health, and body appearance, the rate of satisfaction is also high.
